# Morning Cortisol Levels Affected by Sex and Pubertal Status in Children and Young Adults

**DOI:** 10.4274/Jcrpe.892

**Published:** 2013-05-30

**Authors:** Sarah L. Tsai, Kelly J. Seiler, Jill Jacobson

**Affiliations:** 1 Children’s Mercy Hospital and Clinics University of Missouri Kansas City School of Medicine, Department of Pediatric Endocrinology, Kansas City, MO, USA; 2 Pediatric Endocrinology and Diabetes, Blank Children’s Hospital, Children’s Health Center II, Department of Pediatric Endocrinology and Diabetes, Des Moines, IA

**Keywords:** Morning cortisol, diurnal rhythm, adrenal insufficiency screening

## Abstract

**Objective:** Morning cortisol levels are frequently used as screening tests for adrenal insufficiency in both adults and children. Reports differ on the specificity of this measurement. The present study was undertaken to determine whether sex or pubertal status affected morning cortisol values.

**Methods:** We measured morning cortisol levels and performed low-dose adrenocorticotropic hormone stimulation test in 35 healthy male and female subjects (ages 6-34) ranging in Tanner stage (TS) from TS 1 to TS 5. Testing was initiated at 08:00 after an overnight fast. Morning serum total cortisol, free cortisol, cortisol-binding globulin, estradiol (males and females), and testosterone (males) were obtained.

**Results:** Morning total and free cortisol levels were significantly higher in TS 5 participants than in prepubertal children. Using a morning cortisol of 248 nmol/L todefine a normal value, 19/21(90%) of healthy TS 5 subjects exhibit normal values. In contrast, 0/8 TS 1 healthy subjects exhibited a value greater than 248 nmol/L (p=0.0005). We also observed sex differences in morning cortisol levels in pubertal but not in prepubertal subjects. We observed sex differences in morning cortisol levels in TS 5 individuals.

**Conclusions:** Morning cortisol measurements may be more useful as screening tests for adrenal function in adults than in children. TS and sex may be considered in the decision to screen for adrenal insufficiency using morning cortisol or whether to proceed directly to stimulation testing.

**Conflict of interest:**None declared.

## INTRODUCTION

The signs and symptoms of hypocortisolism are often nonspecific. Thus, the diagnosis of adrenal insufficiency can be challenging. Correctly identifying adrenocortical insufficiency is important, as untreated adrenal insufficiency can be life-threatening. Conversely, inappropriate diagnosis of adrenal insufficiency could lead to unnecessary steroid replacement and eventual iatrogenic adrenocortical suppression.

A good screening test ideally should allow the clinician to identify patients who require further diagnostic testing as quickly and reliably as possible. Serum morning (08:00) cortisol estimations are often used as screening tests, as they are less invasive, and less complex to administer than stimulation testing. The morning cortisol is meant to coincide with an individual’s diurnal peak in serum cortisol at approximately 08:00 (1), and, when above a certain threshold, confirm hypothalamo-pituitary-adrenal (HPA) axis sufficiency.

There is no consensus in the medical literature regarding cut-off values for morning cortisol levels. Clinical signs and symptoms along with other biochemical parameters are important elements of diagnosis and should be taken into consideration when interpreting ambiguous basal levels and/or stimulation testing results.

Upon review of screening tests performed in our pediatric endocrine clinic population, we noted a high incidence of failure to identify adrenal sufficiency using morning serum cortisol levels. These failures appeared to be more common in younger children. Review of the literature failed to reveal any systematic investigations of morning cortisol levels in the pediatric population, with particular emphasis on sex and pubertal status. To our knowledge, this is the first report to stratify serum morning cortisol screening with respect to sex and pubertal status in subjects with concomitant confirmatory adrenocorticotropic hormone (ACTH) stimulation testing.

The central question of the current study was to determine the specificity of serum morning cortisol levels as a screen for adrenocortical sufficiency in healthy children and adults. Additionally, we sought to determine whether serum morning cortisol levels were sexually dimorphic or affected by pubertal status. 

## METHODS

The study population comprised 35 participants (18 males and 17 females). Their mean age was 17.5+8 years (range 6.7-34.3 years). All subjects were determined to be healthy, based on pre-test screening and physical examinations. All Tanner stages (TS) were appropriate for age. Exclusion criteria included acute and chronic moderate-severe illness, use of oral contraceptives, pregnancy, recent use of inhaled, oral, or topical steroids (within the previous 6 weeks), use of performance enhancing over-the-counter medications, or use of any other medications known to affect adrenal steroidogenesis.

The protocol was approved by the institutional review board of Children’s Mercy Hospital and Clinics. Informed consent and assent were obtained prior to testing. Stimulation testing was performed by experienced endocrine nurses. All subjects fasted overnight. The low-dose ACTH stimulation test was performed in all subjects using 1 mg cosyntropin (Cortrosyn, Amphastar Pharmaceuticals, Rancho Cucamonga, CA). No stock solutions were used, and the dilution was carried out on each testing day to a final concentration of 1 µg/mL in a total volume of 1.0 mL diluted in 0.9% normal saline. Testing was initiated at 08:00. Blood samples were drawn from an i.v. cannula at 0, and 10, 20, and 30 min after cosyntropin injection for determination of total cortisol. Free cortisol was measured at 0 and 30 min after injection. Additionally, estradiol (males and females), testosterone (males), and cortisol-binding globulin (CBG) were measured at baseline. All post-menarcheal females were required to undergo urine pregnancy screening. All participants older than 18 years of age were presumed to be TS 5 for pubertal development.

Two male subjects underwent repeat testing because of missing time points. For these subjects, the results of the repeat (complete) testing were used.

For the low-dose ACTH stimulation test, a normal response was defined as a peak serum cortisol concentration of equal to or greater than 495 nmol/L (2). Any subject failing to mount a cortisol response of 495 nmol/L subsequently underwent high-dose (250 µg) ACTH stimulation testing on a different testing day. For this test, cortisol was measured at 0 and 60 min after ACTH injection. A normal response was again defined as a cortisol level of equal to or greater than 495 nmol/L.

Total and free serum cortisol levels were measured by liquid chromatography/tandem massspectrometry and equilibrium dialysis/radioimmunoassay, respectively (Quest Diagnostics-Nichols Institute, San Juan Capistrano, CA). The lower limit of detection (LOD) for total cortisol was 1.1 nmol/L. The interassay coefficients of variation were 7.6%, 4.8%, and 6.6% for low, medium, and high cortisol values, and intraassay coefficients of variation were 3.8%, 3.0%, and 4.6% for low, medium, and high cortisol levels. The lower limit of detection for free cortisol was 0.8 nmol/L.

For low free cortisol levels, the interassay and intraassay coefficients of variation were 14.7% and 9.8 %, respectively. For high free cortisol levels, the interassay and intraassay coefficients of variation were 13.1% and 8.2 %, respectively. CBG was measured by radioimmunoassay (Quest Diagnostics-Nichols Institute, San Juan Capistrano, CA).

Serum estradiol and testosterone were measured by radioimmunoassay (test kits from Diagnostic Products Corporation, Los Angeles, CA) in the clinical endocrinology laboratory of Children’s Mercy Hospital and Clinics. The LOD for estradiol was 5 pg/mL with interassay and intraassay coefficients of variation of 5%. The LOD for testosterone was 4 ng/mL. The interassay coefficients of variation of 12% and 6% at low and high testosterone levels, and the intraassay coefficient of variation was 5%.

## STATISTICAL ANALYSIS

All of the hypotheses involving group comparisons were evaluated using ANOVA. Because cortisol is secreted in a pulsatile fashion, data were not always normally distributed. Therefore, log cortisol and log free cortisol values were used for all comparisons. Correlation coefficients were calculated by using the log of total and free cortisol levels.The sufficiency/insufficiency rates were compared using the Fisher’s exact test. 

## RESULTS

**Maturational Effects on Morning Total and Free Cortisol Values**

Morning (08:00) total cortisol values were higher in TS 5 individuals compared to TS 1 individuals when data from males and females were combined [log (total cortisol) =2.5±0.13 mmol/L in TS 5 versus 2.3±0.07 mmol/L in TS 1 participants; p=0.0007 (mean±SD)] Morning free cortisol values were also higher in TS 5 individuals compared to TS 1 individuals. Log (free cortisol) was 1.6±0.19 mmol/L in TS 5 versus 1.2±0.29 mmol/L in TS 1 participants (p=0.0016). Using one published definition of a normal (unstimulated) a.m. cortisol value of 248 nmol/L (3), we observed maturational differences in “normal” values. Zero of 8 TS 1 subjects displayed values >248 nmol/L. In contrast, 19 of 21 TS 5 subjects passed this definition of normal (p=0.0005). As we recruited healthy subjects with no risk factors for adrenal insufficiency, specificity for the 8 am cortisol of 248 nmol/L was 0% in TS 1 individuals and 95% in TS 5 individuals.

**Sex Effects on Morning Total and Free Cortisol Values**

**Figure 1:**
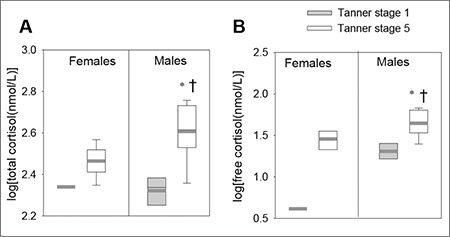
Morning cortisol levels in Tanner stage (TS) 1 and 5 male and female participants. A) total cortisol, and B) free cortisol. A) TS 5 males exhibited higher total cortisol levels compared to Tanner stage 1 males ( p=0.0001) and compared to TS 5 females (p=0.005). B) TS 5 males exhibited higher free cortisols compared to TS 1 males (p=0.0007) and compared to TS 5 females (p=0.018) (TS 1 females n=1; TS 1 males n=5; TS 5 females n=10; TS 5 males n=11)

The data were also analyzed by stratifying TS 1 and TS 5 individuals separately by sex. Figure 1 shows the log of total and free cortisol values in TS 1 and 5 males and females. TS 5 males displayed significantly higher morning total cortisol levels compared to TS 1 males (Figure 1A; *p=0.0001). TS 5 males also displayed significantly higher morning total cortisol levels compared to TS 5 females (Figure 1A; †p=0.005). TS 5 males also displayed significantly higher morning free cortisol levels compared to TS 1 males (Figure 1B; *p=0.0007) and significantly higher morning free cortisol levels compared to TS 5 females (Figure 1B; †p=0.018).

**Relationships Between TS and Total and Free Cortisol Values**

**Figure 2:**
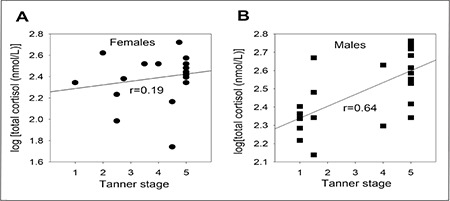
Correlations between Tanner stage and total cortisol levels in pediatric. A) females (r=0.19; p=0.42) and B) males (r=0.64; p=0.001) (n= 17 for female participants and 18 for male participants)

No strong correlation was seen between TS and log total cortisol values in females (Figure 2A; r=0.19; p=0.042). However, a strong direct correlation was seen between TS and log total cortisol values in males (Figure 2B; r=0.64; p=0.001). A formula could be derived for the relationships between TS and total cortisol values in males, as follows: log (total cortisol) =0.07 (TS) + 2.3.

**Figure 3:**
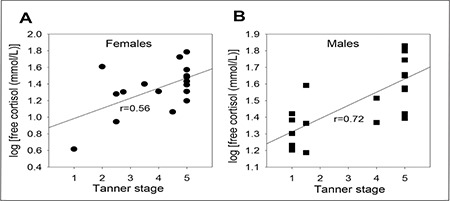
Correlations between Tanner stage and free cortisol levels in pediatric A) females (r=0.56; p=0.18) and B) males (r=0.72; p=0.002) (n=17 for female participants and 18 for male participants)

Figure 3A shows the TS versus log of free a.m. cortisol in females. Figure 3B shows the TS versus log a.m. free cortisol in males. Strong direct correlations were also seen between TS and log total free values in both females and males (Figure 3A, r=0.56, p=0.018 in females; Figure 3B, r=0.72, p=0.0003 in males). Formulae could be derived for the relationships between TS and free cortisol values. In females: log (free cortisol) = 0.32 (TS) + 0.86. In males: log (free cortisol) = 0.08 (TS) + 1.2.

**Maturational and Sex Effects on ACTH Stimulation Testing**

No maturational differences were seen in delta cortisol values, peak cortisol values, free cortisol values, or delta cortisol values in the ACTH stimulation testing. No sex differences were observed in peak total or free cortisol or in incremental rise of total or peak cortisol (data not shown).

**Maturational and Sex Effects on CBG**

No maturational or sex differences were seen in CBG levels. Males exhibited CBG levels of 30.9±4.9 mg/mL and females exhibited CBG levels of 30.3±4.0 mg/mL (mean±SD; p=0.7). TS 1 participants exhibited CBG levels of 29.6±5.6 mg/mL and TS 5 participants CBG levels of 31.2±4.9 mg/mL (p=0.5).

**Specificity of Low-Dose ACTH Stimulation Testing**

Utilizing a cortisol level of 495 nmol/L to define a passing level for the low-dose ACTH stimulation test (2), 31 out of 35 (89%) of our population of healthy subjects exhibited adequate cortisol responses. As we recruited healthy subjects with no risk factors for adrenal insufficiency, specificity for this test is 89%. No sex differences or maturational differences in pass rate were observed. Utilizing a stimulated cortisol level of 415 nmol/L, 33/35 subjects passed, yielding a specificity of 94%.

All 4 subjects who failed low-dose ACTH stimulation testing underwent repeat testing with high-dose ACTH. One subject passed with a peak cortisol of 660 nmol/L. Three subjects, including two sisters, displayed subnormal responses to high-dose ACTH stimulation tests. These subjects peaked at 440 nmol/L, 440 nmol/L and 468 nmol/L. The subjects denied any exposure to glucocorticoids or steroid hormones. A thorough endocrine work up ruled out congenital adrenal hyperplasia and failed to reveal any other etiology for adrenal insufficiency. Data from the subjects in mid-puberty were not included in the analyses of sex differences or maturational differences, as only TS 1 and TS 5 subjects were utilized for these analyses. 

## DISCUSSION

This current study demonstrates both maturational and sex differences in morning cortisol measurements. These maturational and sex differences may have implications for clinical practice when using morning serum cortisol as a screen for HPA axis function.

Our study suggests that morning cortisol levels are less likely to confirm adrenal sufficiency in prepubertal individuals compared to sexually mature individuals. An additional finding is sex differences in the sexually mature participants: morning cortisol levels are significantly higher in TS 5 males than in TS 5 females.

The maturational and sex differences in cortisol levels do not appear to relate to maturational or sex differences in CBG because free cortisol levels parallel the total cortisol levels. Furthermore, no maturational or sex differences were seen in CBG levels. In contrast to our findings with unstimulated 08:00 cortisol levels, our data revealed no major maturational or sex differences in cortisol responses to low-dose ACTH stimulation testing.

The main limitation of this study is its small sample size, with study participant numbers becoming smaller when data are grouped by sex and sexual maturity.

Our work is the first in children that has systematically examined the relationships among serum cortisol, sex, and pubertal status. All of our participants were clinically examined in order to ensure accurate Tanner staging. Furthermore, all participants underwent stimulation testing to further determine the functionality of the HPA axis.

Some studies have advocated the use of morning cortisol levels for screening HPA axis in children and adults. Morning plasma cortisol levels above 524 nmol/L or below 83 nmol/L can possibly serve as reasonable predictors of normal, or abnormal HPA function, respectively (4). Clinically, however, most a.m. cortisol levels fall intermediate to these levels. In one study, a passing level for basal cortisol was 248 nmol/L (3). Others have indicated that levels above 420 to 450 nmol/L indicated healthy HPA axes (5,6). Tordjman states that a basal cortisol of 276 nmol/L or greater is commonly felt to indicate a normal HPA axis (4). Yet, in a group of participants with known impaired HPA function based on hypoglycemia or metyrapone, the average morning cortisol was 306 nmol/L (2). A meta-analysis of pediatric data from three studies (211 children) indicated that if the morning cortisol is >415 nmol/L, then adrenal insufficiency is unlikely. In contrast, if the morning cortisol is ≤88 nmol/L, then adrenal insufficiency is likely to be present (7). If a patient’s morning cortisol level is in the 88-415 nmol/L range, a need for further diagnostic testing is indicated. Exactly what the designated cut-off value should be for children and adults has not been determined conclusively in the medical literature. It is important to take individual biochemical and clinical features into consideration when determining whether further diagnostic testing is needed.

We have compared our study with previous studies examining maturational effects. A study examining diurnal salivary cortisol rhythms in Tsimane’Amazon foragers demonstrated strong age effects across human development, with basal levels and slopes increasing into adulthood and flattening over age 60. The observations in this population were different from industrialized populations in that they revealed lower HPA activity overall. Therefore, not all conclusions may be appropriate for generalization to the industrialized world (8). An examination of diurnal salivary monitoring in school-aged children revealed that cortisol concentrations were higher in 9-year-old children compared to 6-7-year-old children (9). Age-matched postmenarcheal girls exhibited higher cortisol levels than premenarcheal girls, but this was only seen in the evening. Additional studies (10,11) have demonstrated higher cortisol concentrations in postmenarcheal females irrespective of sampling time (morning, midday, evening) and have found statistically significant differences in salivary cortisol concentrations at different pubertal stages. However, pubertal status was estimated based on age alone and not on Tanner staging by clinical examination, as it was in our study.

Rosmalen et al (12) found no significant correlations between TS and cortisol level for all subjects, or for males and females separately. Of note, pubertal stage was determined by parent interview using schematic drawings. This method may be inaccurate, particularly for boys, when the only sign of puberty at age 10-12 may be a modest increase in testicular volume. This study found no significant correlation between BMI and cortisol levels.

We have compared our study with previous studies in the literature examining the effects of sex on cortisol levels. Interestingly, Klimes-Dougan et al (13) demonstrated sex differences in cortisol levels among adolescents and reported higher values in females at midday and later afternoon. In another study (9), there were no sex differences in the median cortisol values for all children ages 6-18 years, and median cortisol values from different times of the day were used.

Rosmalen et al (12) conducted a large study on Dutch children (ages 10-12) and found that salivary cortisol levels differed between boys and girls in the morning but not in the evening. Sex differences were already present in prepubertal children and significant differences were found between boys and girls in TS 1, with girls having higher a.m. cortisol levels. Other factors have been shown to affect cortisol levels in children, such as weight, insulin sensitivity, circadian rhythms, transcortin levels, sleep-wake cycles and stress factors like anxiety and depression (14,15,16,17,18). The relationship between individual cortisol variability and psychosocial factors is likely an important moderating factor in HPA functioning that is not fully understood.

Metabolic factors such as obesity, glucose tolerance and metabolic syndrome are well known to affect morning cortisol levels. Weigensberg et al (19) found that serum morning cortisol levels were higher in overweight Latino youths with metabolic syndrome, and that those with impaired fasting glucose had significantly higher morning cortisols than those who had normal fasting glucose. Salivary cortisol levels have been shown to be increased in obese children with anxiety or depression compared to obese children without affective morbidity. This indicates that the emotional milieu of an obese child is likely to further affect the HPA axis (20).

Future research direction could include larger scale studies of morning cortisol levels in children throughout various stages of puberty. Our study suggests that type 2 statistical errors may occur, (i.e., real differences are not detected) if cortisol values from pediatric participants are not stratified by both TS and sex. We have provided evidence that serum morning cortisol levels may not be as clinically useful in assessing adrenal function in prepubertal children compared to older children. Further, morning cortisol levels may not be as helpful in females compared to males in assessing adrenal function, regardless of sexual maturity state. Both TS and sex should be taken into account when interpreting screening tests clinically. In addition, TS and sex need to be considered in the decision to screen for adrenal insufficiency using morning cortisol or whether to proceed directly to stimulation testing.

**Acknowledgements**

We appreciate the assistance of the endocrinology nurses Monica Cameron, Tamra Radatz, Faith Steeby, and Malisa Putnam.
